# Effect of alcohol addition on the structure and corrosion resistance of plasma electrolytic oxidation films formed on AZ31B magnesium alloy†

**DOI:** 10.1039/d0ra01154a

**Published:** 2020-03-02

**Authors:** Hidetaka Asoh, Kento Asakura, Hideki Hashimoto

**Affiliations:** Department of Applied Chemistry, Kogakuin University 2665-1 Nakano, Hachioji Tokyo 192-0015 Japan asoh@cc.kogakuin.ac.jp

## Abstract

The effect of the addition of alcohol to a Na_3_PO_4_-based electrolyte on plasma electrolytic oxidation (PEO) of AZ31B magnesium alloy was investigated. Anodization with spark discharge was conducted in Na_3_PO_4_-based electrolyte containing various alcohols (*e.g.*, ethanol, ethylene glycol, and glycerol) at a constant current density of 200 A m^−2^ and a constant temperature of 25 °C. Voltage–time curves during the PEO process, the film structure, surface roughness, crystallographic structure, composition, corrosion resistance, and withstand voltage were investigated using various analytical equipment and electrochemical measurements. When the electrolyte containing alcohol was used, the initial bending voltage was higher than that observed using the basic electrolyte without alcohol addition, as was the oscillation voltage during the PEO process. For a given amount of electricity supplied, the addition of alcohol into the basic electrolyte tended to increase the thickness and corrosion resistance of PEO films formed while effectively reducing surface roughness. In particular, the addition of a polyhydric alcohol (*i.e.*, ethylene glycol and glycerol) could act not only as a leveler for the formation of compact film but also as an enhancer for film qualities, such as corrosion resistance and withstand voltage. The patterns observed for Na_3_PO_4_-based electrolyte containing alcohol also hold for Na_2_SiO_3_-based electrolyte containing alcohol.

## Abstract 2

Alcohol used as an additive could act not only as a leveler for the formation of compact film but also as an enhancer for film qualities, such as corrosion resistance and withstand voltage.

## Graphical abstract

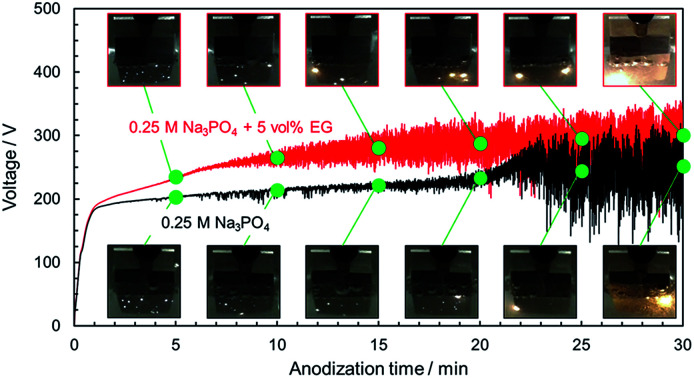

## 1 Introduction

Magnesium and its alloys have attracted attention as lightweight materials with various advantages, such as low density, high strength-to-weight ratio, and substantial recyclability. While such advantages have attracted the interest of researchers and engineers in various fields, including automotive, electronic, and medical devices, drawbacks remain, in particular, poor corrosion resistance. To improve corrosion resistance, alloying magnesium with other elements and/or various surface treatments (*e.g.*, chemical conversion, electroless plating, and anodization) have been studied in recent decades.^1–6^

Among several types of surface treatments, anodization with spark discharges at a high voltage (so-called plasma electrolytic oxidation (PEO) or micro-arc oxidation (MAO)) is expected to improve the corrosion resistance of magnesium and its alloys.^7–9^ In the PEO process for magnesium alloys, alkaline solutions containing phosphate,^10–13^ silicate,^14–16^ mixtures of phosphate and silicate,^17–19^ or fluoride^20–23^ are generally used. In recent years, the PEO process in an electrolyte containing various particles as additives has also been studied extensively as an approach for improving film properties.^24–26^ When corrosive solutions penetrate in the structural defects (including pores and cracks) of the PEO films, the localized corrosion of the substrate occurs. Therefore, the PEO films having a compact barrier-type passive layer give excellent corrosion resistance. In particular, the PEO process in an electrolyte containing various additives provides a beneficial impact on corrosion resistance due to reduction in coating porosity as well as increase in compactness. For details of corrosion resistance of the PEO films, see review papers.^8,9^

Recently, we investigated the microstructures and corrosion resistance of the anodic oxide films formed on AZ31B magnesium alloy by direct current (DC) anodization under continuous sparking in alkaline phosphate electrolyte.^27–29^ In our previous studies, we found that the microstructures (*e.g.*, diameter and number of pores/spherical cavities) of the anodic films formed under sparking were strongly affected not only by the formation voltage itself but also by the degree of sparking, in particular, the size, number, and appearance frequency of sparks.^27^

In general, corrosion and wear resistance of PEO films are improved by the hard, ceramic-like coatings several tens of microns in thickness. Therefore, the formation of such PEO films requires voltage and current considerably higher than those required in conventional DC anodization without sparking. In addition, corrosion resistance is considered difficult to ensure *via* the PEO process alone. Therefore, the improvement of corrosion resistance by secondary treatments, such as a two-step PEO^30,31^ and post-sealing treatment,^32–34^ are also being studied. In other words, further fundamental study is needed to develop a PEO process with lower energy consumption, low cost, and high efficiency.

In the present study, the effect of electrolysis conditions on the electrochemical formation behavior, microstructure, thickness, composition, and corrosion resistance of anodic films formed on AZ31B magnesium alloy was investigated with a focus on the addition of alcohols to the electrolyte. This focus has been relatively little studied compared with the numerous PEO studies using fluoride or particles as additives,^20–26^ especially with respect to the properties of the formed films. We have previously reported on anodization of magnesium in an electrolyte containing ethylene glycol, noting that organic species, unlike simple aqueous electrolytes, have a positive effect on corrosion resistance.^35^ Moreover, in recent years, we found that the addition of alcohol into an electrolyte improves the formation efficiency of anodic alumina on aluminum during mild anodization.^36,37^ Based on this knowledge, we focused on the effect of alcohol as an additive and investigated the influence of electrolyte composition on the properties of PEO films. Sodium phosphate (Na_3_PO_4_), which is generally used for the PEO process, was used as the main electrolyte, whereas sodium silicate (Na_2_SiO_3_) was used for comparison. Corrosion resistance and passivity were evaluated by simple salt immersion tests and voltage sweeps instead of typical electrochemical tests (*e.g.*, potentiodynamic polarization and electrochemical impedance spectroscopy measurement using a three-electrode system).

## 2 Experimental

### 2.1 Specimens and PEO process

An extruded plate of AZ31B alloy with a thickness of 1 mm was cut into pieces to give a working area of 5 cm^2^ to be used as specimens. The chemical composition of the alloy was the same as previously used.^29^ Prior to PEO treatment, the specimens were degreased in acetone in an ultrasonic bath for 3 min and immersed in a mixed acid solution of 10 vol% HNO_3_ and 2 vol% H_3_PO_4_ for 20 s at room temperature.

DC anodization was conducted mainly in 0.25 mol dm^−3^ Na_3_PO_4_ or Na_2_SiO_3_ containing alcohol, at a constant current density of 200 A m^−2^ at 25 °C. A stainless steel sheet was used as a cathode. Various alcohols, such as ethanol (abbreviated EtOH), ethylene glycol (EG), and glycerol (GLY), were used as additives without further purification. To clarify the effect of alcohol itself on the PEO process, other additives, such as fluoride, were not added to the basic electrolyte. As a reference, the single electrolyte without the addition of alcohol and 0.5 mol dm^−3^ Na_3_PO_4_ were also used for the PEO process.

### 2.2 Characterization

The morphology of anodic film obtained by the PEO process was evaluated using field-emission scanning electron microscopy (FESEM, JEOL JSM-6701F) and three-dimensional laser scanning microscopy (KEYENCE VK-X200). The thickness of the anodic films was measured using an eddy-current coating-thickness tester (Kett Electric Laboratory LH-373). The average value of 20 point measurements was used. The crystallographic structure of PEO films was evaluated by X-ray diffractometry (XRD, Rigaku MiniFlex600). Glow discharge optical emission spectroscopy (GD-OES, Jobin-Yvon JY5000RF) was used for measuring the depth profiles of constituent elements in the films accompanied by argon ion sputtering.

The corrosion resistance of PEO films was evaluated using an immersion test in a 0.1 mol dm^−3^ NaCl aqueous solution at 30 °C for 100 h, as described elsewhere.^29^ The passivity (the effectiveness of the insulation) of anodized specimens was evaluated by voltage sweeping of the specimen at 1 V s^−1^ in 0.25 mol dm^−3^ Na_3_PO_4_. Because solvent action induces a more complex situation in the case of electrolytes that dissolve the oxide (*e.g.*, acid or neutral solution), 0.25 mol dm^−3^ Na_3_PO_4_ was used as the test solution. Voltage sweeping was conducted using DC power supply (Takasago GP0500-1R) and sweep adapter (Takasago AP-4KS). In all cases, the test solution, exposed to ambient air, was thermostatically controlled at 25 °C. Here the current–voltage (*I*–*V*) characteristic for anodized specimens was defined as the relationship of the measured leakage current through the PEO films to the given voltage.

## 3 Results and discussion

### 3.1 Voltage–time curves during the PEO process in Na_3_PO_4_-based electrolytes

**Fig. 1:**
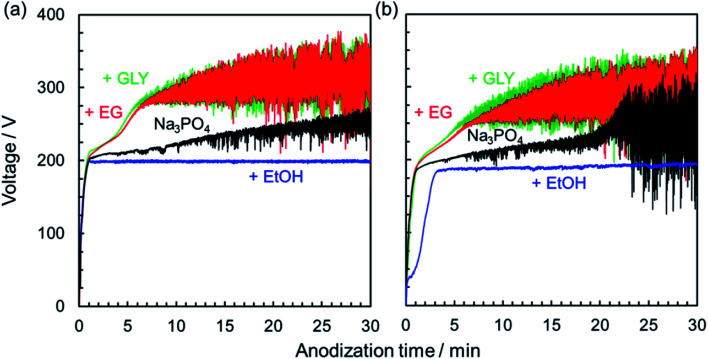
Voltage–time curves for the anodization of (a) 99.95% magnesium and (b) AZ31B magnesium alloy in 0.25 mol dm^−3^ Na_3_PO_4_ with 5 vol% various alcohols under a constant current density of 200 A m^−2^ at 25 °C.

Fig. 1 presents typical voltage–time (*V*–*t*) curves for constant current density anodization of AZ31B magnesium alloys at 200 A m^−2^ in Na_3_PO_4_ solutions with and without different types of alcohol as an additive. To investigate the influence of the purity of magnesium substrate on the PEO process, *V*–*t* curves for 99.95% magnesium are shown for comparison (Fig. 1a).

At an initial stage of anodization (∼1 min), the voltage increased almost linearly, suggesting a formation of a compact oxide film with electrical resistance. *V*–*t* curves bent at around 200 V; 200 V was defined as the bending voltage (*i.e.*, the first breakdown voltage). At this time, the film growth process changed from the formation of a barrier-type oxide film to a porous-type film and PEO film, as previously reported.^29^ Subsequently, voltage increased gradually with voltage oscillation. Fundamental variation of voltage was basically similar regardless of the purity of magnesium substrate.

In the case of electrolyte containing EtOH as an additive, no obvious increase in voltage was observed after the *V*–*t* curve bent at around 200 V. The electrolyte containing EtOH was not suitable for film formation probably due to slightly low pH (pH = 12.65) and weak base compared with that containing Na_3_PO_4_ alone (pH = 12.73). It is well known that the dielectric constant of the medium plays an important role, controlling the properties of the solution, such as conductivity and degree of dissociation.^38,39^ Because the dielectric constant of an alcohol–water mixture generally decreases with increasing alcohol content,^38^ the dissociation of electrolyte might be suppressed by the addition of EtOH. That is, this result indicates that a certain concentration of dissociated ions is necessary to start the growth of an anodic oxide film through the PEO process.

On the other hand, when EG or GLY was used as an additive, the rates of the increase of voltage and attainable voltage were higher than when Na_3_PO_4_ was used without alcohol. Ionic conductivity might be reduced by the addition of alcohol in the Na_3_PO_4_-based electrolyte. In other words, the electrolyte resistance was increased simply by the viscosity contribution of added EG or GLY.

**Fig. 2:**
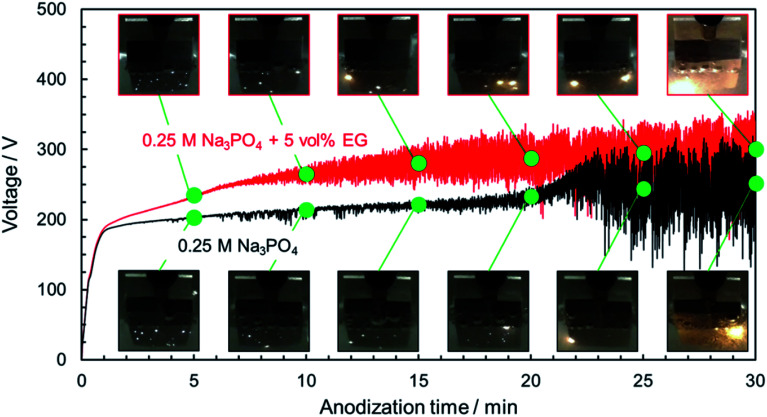
Photographs showing the change in surface appearance of AZ31B specimens during the PEO process, corresponding to marked points in *V*–*t* curves. Anodization conditions were the same as those in Fig. 1(b).

Anodization of magnesium alloy with spark discharge is considered to be divided into three or four stages: normal anodization, spark anodization, micro-arc oxidation, and arc oxidation.^9,40–42^ This behavior is similar to that observed in the PEO treatment of aluminum.^43^ To analyze in greater detail the *V*–*t* response and phenomena occurring on the magnesium substrate surface during the PEO process, the relationship between the *V*–*t* curves and discharge characteristics observed on the specimen surface was investigated. Fig. 2 presents the change in the surface appearance of AZ31B magnesium alloys during the PEO process, corresponding to marked points on *V*–*t* curves. In the case of a 0.25 mol dm^−3^ Na_3_PO_4_ solution, the voltage increased almost linearly at the initial stage. After the *V*–*t* curve bent at around 190 V, voltage oscillation was observed. Voltage increased gradually up to approximately 210 V after 20 min. During this stage, many small, bright sparks appeared and moved along the surface.

After PEO treatment for 20 min, strong voltage oscillation with intense sparking discharge and gas evolution began. During this stage, the small white sparks turned into large orange sparks, and sparks were localized at the specimen edge. The spark size and strength increased, whereas the number of sparks decreased with time.

In the case of 0.25 mol dm^−3^ Na_3_PO_4_ with 5 vol% EG, the rate of voltage increase was higher than that for Na_3_PO_4_ alone in the region above 190 V. Strong voltage oscillation began earlier than that for Na_3_PO_4_ alone. If a specific thickness (or a specific oxide resistance) is necessary to initiate voltage oscillation, the addition of EG is thought to be effective in forming a compact oxide with higher resistance at an earlier stage. Moreover, sparking was distributed more widely than in the case of Na_3_PO_4_ alone. Although intense voltage oscillation occurred in the region after 10 min, the orange sparks moved slowly across the surface. It is clear that the addition of alcohol into electrolyte affects the behavior of film formation during the PEO process.

### 3.2 PEO film thickness, surface morphology, and roughness

**Fig. 3:**
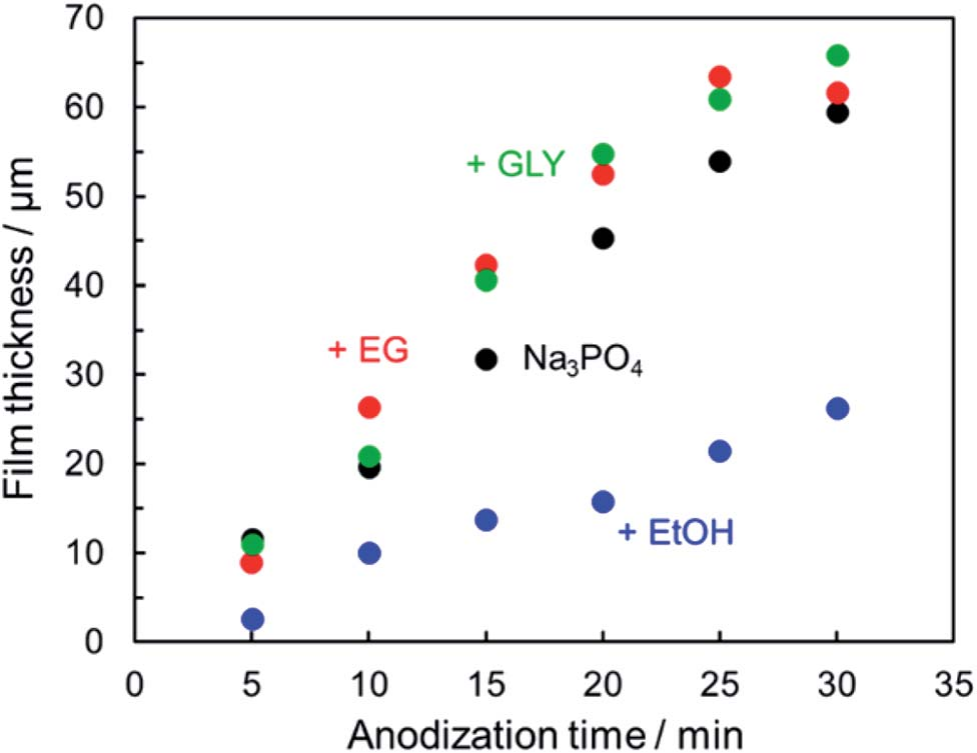
Thickness of the resulting PEO film as a function of anodization time. Anodization conditions were the same as those in Fig. 1(b).

Fig. 3 presents the relationship between film thickness and reaction time. Corresponding data with error bar are shown in the ESI (Fig. S1†). In the case of DC constant-current anodization, the thickness of the anodic oxide film is known to be determined predominantly by the amount of electric charge (*i.e.*, the anodization time at constant current). Even in the present PEO process, it was confirmed that the relationship between film thickness and anodization time was approximately linear in all cases, although the slope of the linear trend varied depending on the type of electrolyte. The proportionality constant of film thickness for a given reaction time, *i.e.*, the growth rate of the anodic films, was approximately 2 μm min^−1^.

In the case of ethanol addition, however, the growth rate of the anodic film was less than 1 μm min^−1^. Within the present experimental conditions, the growth rate in Na_3_PO_4_ with EtOH was lower than in Na_3_PO_4_ with EG or GLY. The relatively low efficiency of film formation in 0.25 mol dm^−3^ Na_3_PO_4_ with 5 vol% EtOH is attributable mainly to low alkaline phosphate level as described above. In the case of 0.25 mol dm^−3^ Na_3_PO_4_ with 5 vol% EG (pH = 12.73) and GLY (pH = 12.70), electrolyte pH was almost the same as that of 0.25 mol dm^−3^ Na_3_PO_4_ (pH = 12.73), and the growth rate was equal to or greater than that for Na_3_PO_4_ alone. PEO films formed under present conditions exhibited grayish appearance. In particular, PEO films formed in Na_3_PO_4_ with GLY dark gray (see Fig. 10).

**Fig. 4:**
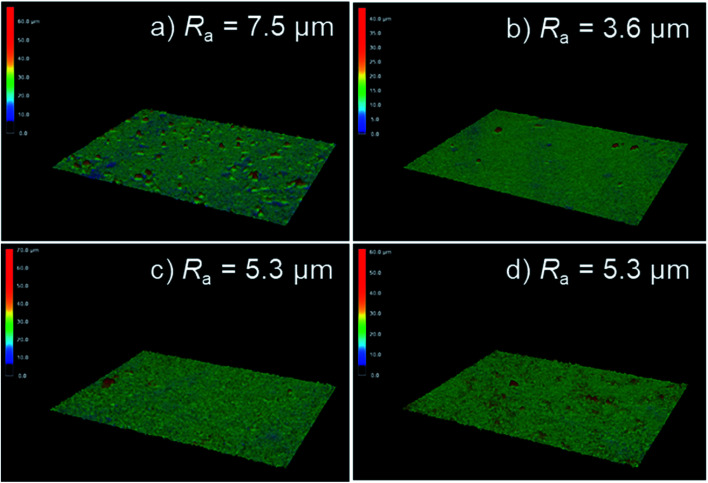
3D laser microscope images of anodized AZ31B specimen surfaces prepared in 0.25 mol dm^−3^ Na_3_PO_4_ with 5 vol% various alcohols under a constant current density of 200 A m^−2^ at 25 °C for 5 min: (a) Na_3_PO_4_ without addition of alcohol, (b) Na_3_PO_4_ with 5 vol% EtOH, (c) 5 vol% EG, and (d) 5 vol% GLY.

The effect of electrolyte composition on surface roughness was also evaluated using three-dimensional laser scanning microscopy. Fig. 4 presents the surface laser microscope images obtained after anodization for 5 min in 0.25 mol dm^−3^ Na_3_PO_4_ with 5 vol% various alcohols. The addition of alcohol led to an obvious change in the surface morphology of PEO films. The arithmetic average roughness *R*_a_ of the film formed in Na_3_PO_4_ was 7.5 μm, as presented in Fig. 4a. The *R*_a_ for the film formed in Na_3_PO_4_ containing EtOH was the least rough, at 3.6 μm, but this is probably because the film itself was thin, as presented in Fig. 3. On the other hand, the films formed in Na_3_PO_4_ with EG or GLY showed lower *R*_a_ even at similar film thicknesses and higher voltage conditions than that in Na_3_PO_4_ alone at the same anodization duration (Fig. 4c and d). Although the spark discharge was expected to be intense due to the high voltage reached, it was actually possible to form a relatively compact film with a low surface roughness. This is thought to be due to the improvement of spark dispersion by the addition of alcohol into Na_3_PO_4_-based electrolyte, especially highly viscous alcohols (*i.e.*, EG and Gly).

**Fig. 5:**
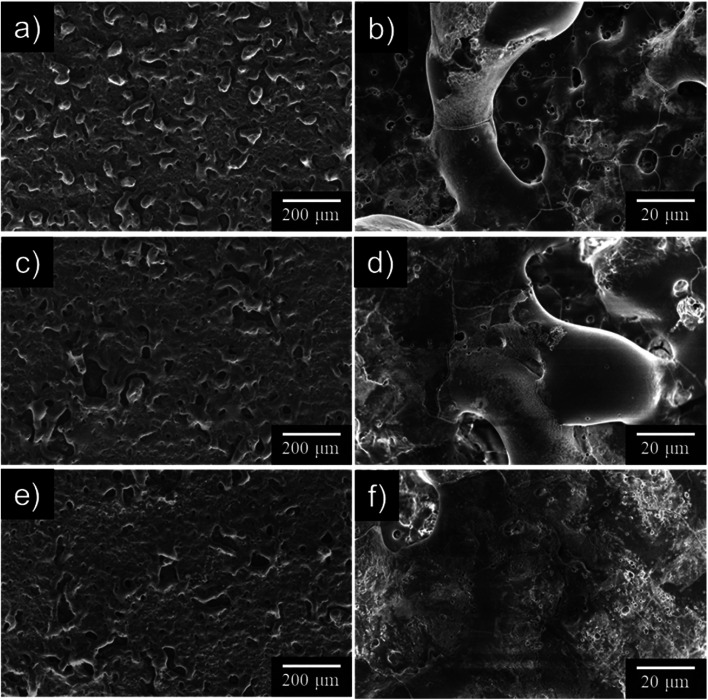
SEM images of anodized AZ31B specimen surfaces prepared in 0.25 mol dm^−3^ Na_3_PO_4_ with different additives under a constant current density of 200 A m^−2^ at 25 °C for 10 min: (a and b) no addition, (c and d) 5 vol% EG, and (e and f) 5 vol% GLY. (b, d and f) High-magnification images of each specimen.

Fig. 5 presents surface SEM images obtained after anodization for 10 min in 0.25 mol dm^−3^ Na_3_PO_4_ with 5 vol% various alcohols. Numerous pores and cracks were observed at the surface of the uneven PEO films. The characteristic film structure implies that the PEO film was formed in a molten state caused by plasma and solidified after rapid cooling, as reported by many researchers. In the case of Na_3_PO_4_ alone, surface roughness was higher than that for other anodization conditions. This result was consistent with the surface roughness trend presented in Fig. 4.

Surface morphology was strongly affected by electrolyte composition. As seen in Fig. 5c–f, the number of protrusions, pores, and cracks decreased with the addition of alcohols, especially GLY. Because numerous intense sparks were distributed over the entire surface of the specimen, PEO films could be formed with smoother surfaces and with fewer voids and cracks than those obtained for Na_3_PO_4_ alone. Concerning the addition of GLY, Wu *et al.* reported a similar leveling effect for the PEO of AZ91D magnesium alloy in Na_2_SiO_3_–NaOH–Na_2_EDTA electrolyte with GLY addition.^44^ They argued that the addition of GLY into silicate electrolyte leads to increase of the unit-area adsorptive capacity of the negative ions at electrolyte/anode interface and thus improves the compactness and corrosion resistance of the PEO films.^44^ Qiu *et al.* also studied the PEO of ZK60 magnesium alloy in Na_2_SiO_3_–KOH–NaF electrolyte with GLY addition.^45^ The results of molecular dynamics simulations showed that GLY molecule adsorbed at the surface of the PEO films and could decrease the number of cracks and increase the thickness of the PEO films. Moreover, they reported that the active sites on the surface during the PEO process are divided into smaller sizes leads to the decrease of the volume of oxygen bubble.^45^ Because the composition of the electrolyte in their studies was complex, however, it is difficult to extract a simple explanation of the effect of alcohol as an additive.

**Fig. 6:**
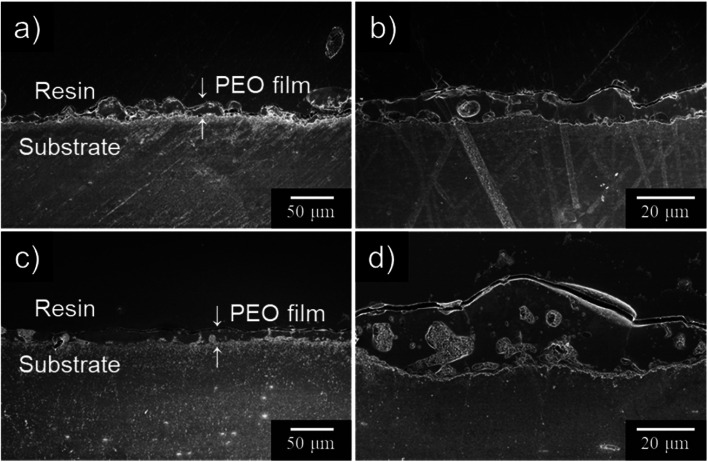
Cross-sectional SEM images of anodized AZ31B specimens prepared in 0.25 mol dm^−3^ Na_3_PO_4_ with EG addition under a constant current density of 200 A m^−2^ at 25 °C for 10 min: (a and b) no addition, (c and d) 5 vol% EG.

Cross-sectional SEM images of the specimens after PEO treatment for 10 min are presented in Fig. 6. When PEO was conducted in 0.25 mol dm^−3^ Na_3_PO_4_ alone, the resulting PEO film thickness was uneven. The spatial heterogeneity of the film thickness is thought to be due to the manner of discharge and movement of sparks. This tendency was consistent with the results of Fig. 3 and 4. On the other hand, PEO film formed in Na_3_PO_4_ with EG exhibited relatively uniform film thickness (Fig. 6b and d).

### 3.3 XRD measurements

**Fig. 7:**
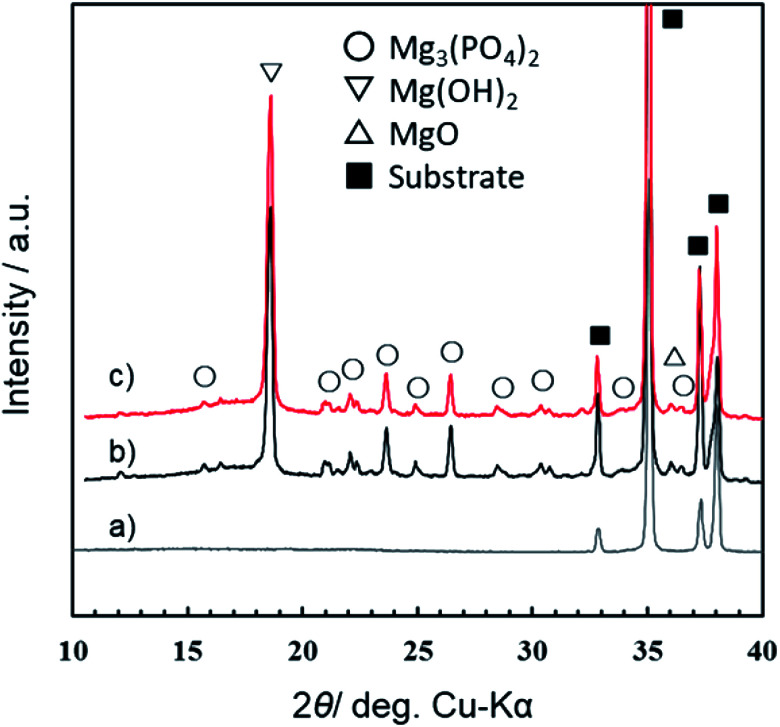
XRD patterns for the specimens after PEO treatment in 0.5 mol dm^−3^ Na_3_PO_4_ with EG addition under a constant current density of 200 A m^−2^ at 25 °C for 10 min: (a) substrate, (b) Na_3_PO_4_ without addition, (c) 5 vol% EG addition.

Fig. 7 presents the XRD patterns of films formed by PEO treatment in 0.5 mol dm^−3^ Na_3_PO_4_ with EG addition under a constant current density of 200 A m^−2^ at 25 °C for 10 min. Here, the concentration of electrolyte was changed from 0.25 mol dm^−3^ to 0.5 mol dm^−3^, which was the standard concentration in our previous studies.^29^ Corresponding data for anodization in 0.5 mol dm^−3^ Na_3_PO_4_ alone were published earlier,^29^ and they are shown is Fig. S2.† Although the concentration of Na_3_PO_4_ used for PEO treatment was different from the standard concentration (0.25 mol dm^−3^) of the present study, the alcohol addition effect on *V*–*t* transient and spark discharge showed almost the same trend as shown in Fig. S2.† Therefore, the influence of the base concentration on the crystallinity and constituent phases of the obtained PEO film is considered to be small. From the XRD results, the diffraction peaks of Fig. 7b and c were almost the same. Both PEO films were composed mainly of Mg(OH)_2_, Mg_3_(PO_4_)_2_, and a small amount of MgO. It is credible that the addition of EG had little influence on the constituent phases and the crystallographic structures of PEO films. For details, see Fig. S3 in the ESI.†

### 3.4 GD-OES measurements

**Fig. 8:**
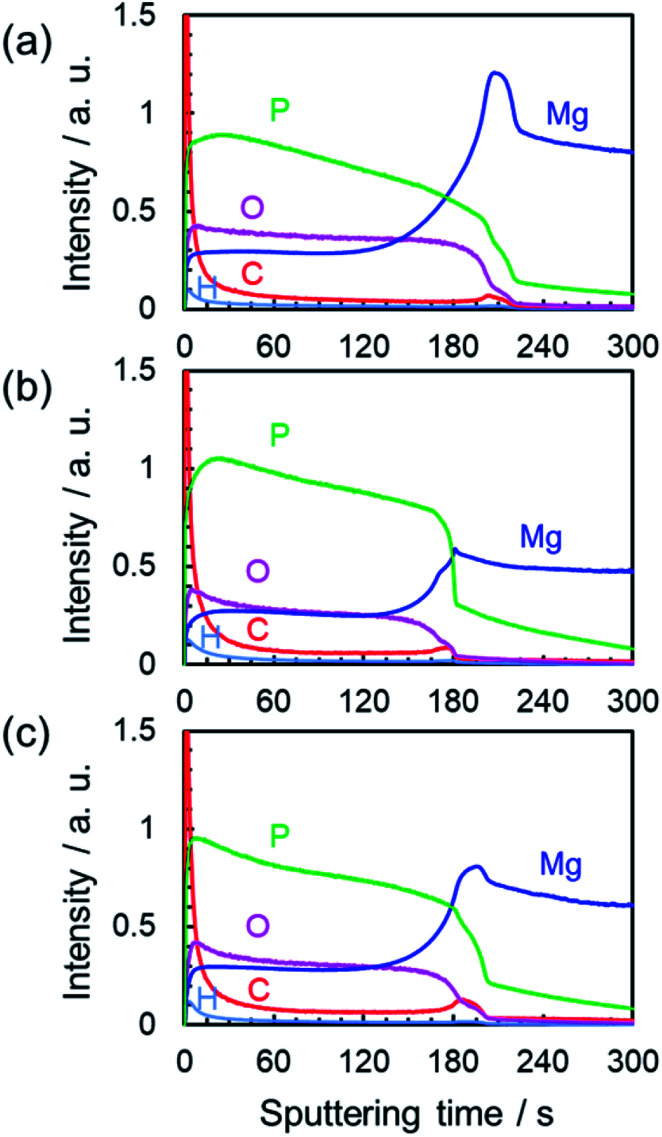
GD-OES depth profiles of constituent elements in the PEO films prepared in 0.25 mol dm^−3^ Na_3_PO_4_ with 5 vol% various alcohols under a constant current density of 200 A m^−2^ at 25 °C for 10 min: (a) Na_3_PO_4_ without addition of alcohol, (b) Na_3_PO_4_ with 5 vol% EG, and (c) 5 vol% GLY.

To evaluate the distribution of constituent elements in the PEO film formed in Na_3_PO_4_ with EG or GLY, the GD-OES depth profiles were measured as shown in Fig. 8. The GD-OES depth profiles for the film formed in Na_3_PO_4_ without alcohol addition are shown for comparison (Fig. 8a). The depth profiles of constituent elements in the film indicate the composition of the film involving substrate-derived element (Mg) and electrolyte-derived P, O and H species. The Mg and O profiles show relatively uniform distributions of these species in the thickness direction while the concentration of P increases toward the outer regions irrespective of the electrolyte composition.

On the other hand, carbon (C) was also detected in the film and seemed to be slightly condensed at the outermost surface and the film/substrate interface in all cases. However, there was little difference between the C profiles in Fig. 8. It should be noted that there is no carbon source in the case of Na_3_PO_4_ alone (Fig. 8a). Therefore, the carbon was considered to be deposited as organic contaminants when surfaces were exposed to electrolyte and room air in all cases. Thus, it was estimated that the addition of alcohol (EG and GLY) had little influence on the composition of the PEO films. This tendency is consistent with the XRD results (Fig. 7 and S3†).

### 3.5 Voltage–time curves during the PEO process in Na_2_SiO_3_-based electrolytes

**Fig. 9:**
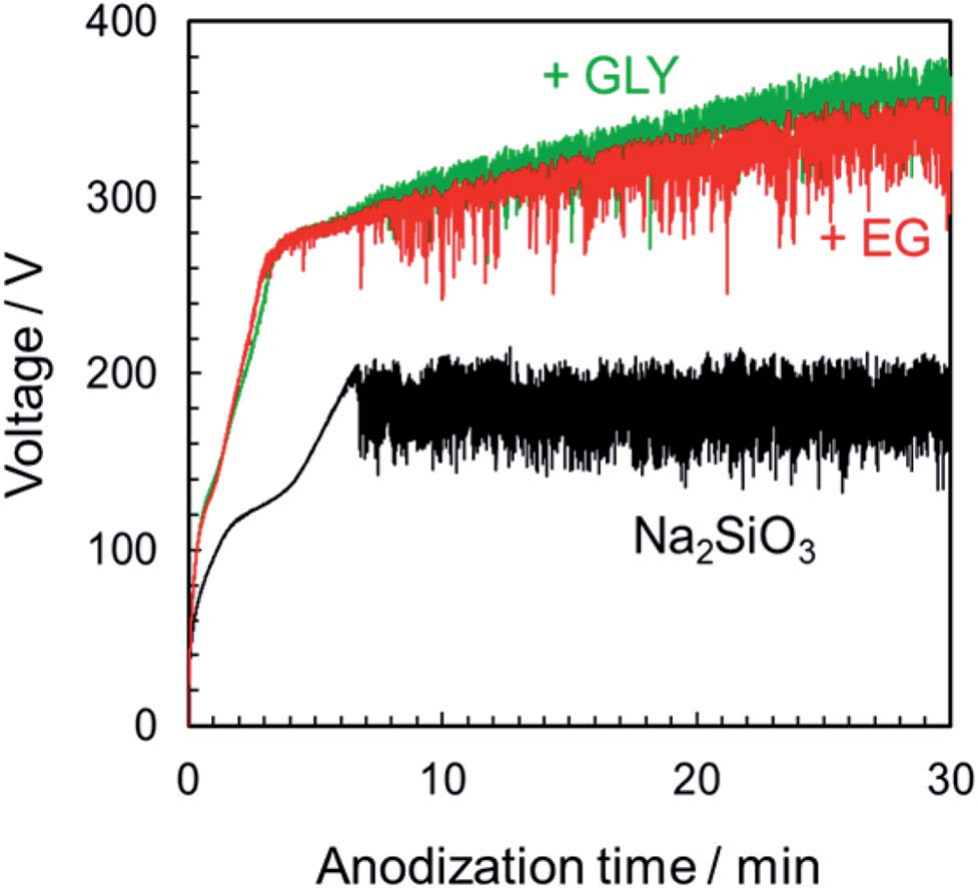
Voltage–time curves for the anodization of AZ31B magnesium alloy in 0.25 mol dm^−3^ Na_2_SiO_3_ with 5 vol% various alcohols under a constant current density of 200 A m^−2^ at 25 °C.

To understand the nature of the effects of basic electrolyte, the basic electrolyte was changed from Na_3_PO_4_ to Na_2_SiO_3_. Fig. 9 presents the *V*–*t* curves in Na_2_SiO_3_ solutions with and without different alcohols as additives. As in Fig. 1b, the voltage increased in the additive system. In the case of Na_2_SiO_3_ alone, the maximum attainable voltage was approximately 200 V. After 7 min, the voltage reached 200 V, and voltage oscillation began. However, because the spark was localized at the edge of specimen, uniform film formation over the entire specimen surface could not be achieved. By addition of alcohol, the voltage increased to approximately 280 V and began to oscillate. Subsequently, the voltage continued to rise slowly as presented in Fig. 9. The PEO film could form uniformly over the entire specimen surface.

### 3.6 Film properties

**Fig. 10:**
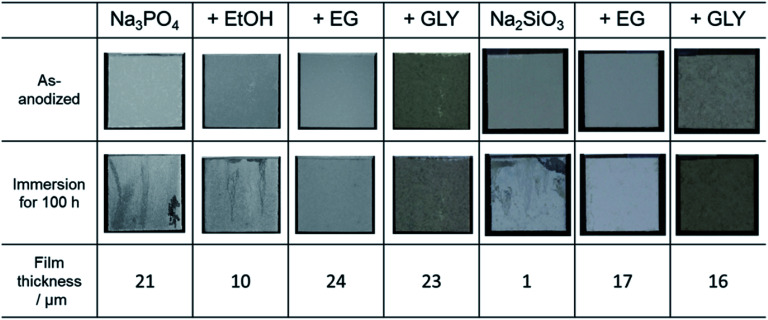
Photographs of anodized AZ31B specimens before and after immersion test for 100 h in 0.1 mol dm^−3^ NaCl aqueous solution. Film thickness of the corresponding PEO film is also shown.

To evaluate the corrosion resistance of anodized specimens, immersion tests were conducted in 0.1 mol dm^−3^ NaCl aqueous solution for 100 h. Here, no posttreatment was applied, *e.g.*, sealing to improve corrosion resistance. Fig. 10 presents the surface appearance of each specimen before and after the immersion test. Discoloration was observed on the specimen surfaces treated in electrolytes without alcohol addition, as well as on those treated in Na_3_PO_4_ with EtOH addition. The insufficient corrosion resistance for the specimens treated in Na_3_PO_4_ with EtOH addition, as well as for those treated in Na_2_SiO_3_ alone, is mainly due to the thin-film thickness. Although the average film thickness of specimens prepared in Na_3_PO_4_ alone was relatively thick at 21 μm, obvious discoloration was observed. Probably the thickness of the PEO film was uneven, as presented in Fig. 6a and b, the corrosion resistance of thin parts of the film being consequently insufficient.

For PEO films prepared in electrolytes with EG and GLY, no discoloration or pitting was observed regardless of the type of basic electrolyte. Corrosion resistance clearly increased with the addition of alcohol. Even in the case of Na_2_SiO_3_ with EG or GLY, specimens exhibited good corrosion resistance despite film thicknesses under 20 μm and without posttreatment, such as sealing. It seems the relatively compact films prevent the penetration of corrosive chemicals (*e.g.*, chloride ion) into the films, acting as protective films with good corrosion resistance.

Corrosion resistance is often evaluated by the corrosion potential or corrosion current density obtained from potentiodynamic polarization curves. Although polarization curves may show the susceptibility of the material to corrosion in designated environments, it is not a suitable method for evaluating the compactness and withstand voltage of PEO films with relatively thick films ranging from several microns to several tens of microns.

To evaluate the corrosion resistance and passivity of such PEO films from a macroscopic point of view, *I*–*V* characteristics measured by the voltage sweep method were analyzed. The principle of investigating the characteristics of oxide coatings is similar to that described in a previous study reported by Hunter *et al.*^46^ According to their report, if any DC voltage less than the anodization voltage is applied, only leakage current will be detected. If any value above the anodization voltage will induce current flow greater than the leakage value. Here, to evaluate the passivity (the effectiveness of the insulation) of anodized specimens, the classical technique was applied.

**Fig. 11:**
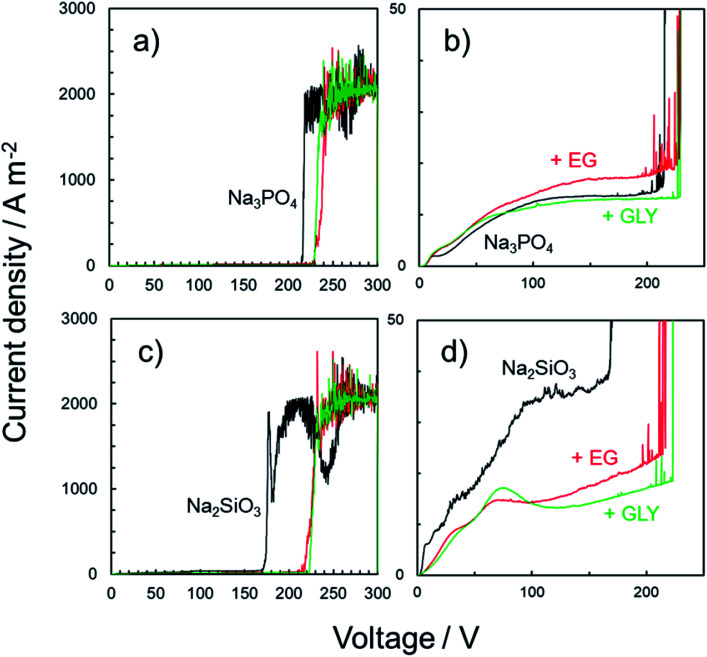
*I*–*V* characteristic curve for the anodized specimens evaluated by voltage sweep. Anodization was carried out in (a and b) 0.25 mol dm^−3^ Na_3_PO_4_ and (c and d) 0.25 mol dm^−3^ Na_2_SiO_3_ with 5 vol% various alcohols under a constant current density of 200 A m^−2^ at 25 °C for 10 min. After PEO treatment, voltage sweep was conducted in 0.25 mol dm^−3^ Na_3_PO_4_ at 1 V s^−1^ up to 300 V at 25 °C. (b and d) Enlarged *I*–*V* curves for each specimen.

Typical current transients obtained for anodized specimens are presented in Fig. 11. The specimens were prepared by PEO treatment for 10 min in various electrolytes with and without alcohol addition. In the *I*–*V* curves obtained by voltage sweep, the withstand voltage was defined as the voltage at the point at which the current increased rapidly (Fig. 11a and c). The withstand voltage provides useful information for the limits of insulation systems of the PEO films depending on the type of electrolyte. Therefore, *I*–*V* characteristics of both PEO films formed in Na_3_PO_4_ and Na_2_SiO_3_ were compared.

In the case of Na_3_PO_4_, relatively low current density of less than 15 A m^−2^ was observed at voltages of up to approximately 210 V (Fig. 11b). An increase in current was observed immediately after reaching 210 V. No such rapid current increase was observed at that juncture for the specimens prepared in Na_3_PO_4_ with EG or GLY. The currents measured at 210 V for both these specimens were less than 20 A m^−2^, which was the same as that for the specimen prepared in Na_3_PO_4_ alone. After reaching 230 V, a rapid current increase was observed for the specimens prepared in Na_3_PO_4_ with EG and GLY. Based on these results, the withstand voltage of specimens prepared in Na_3_PO_4_ with alcohol was approximately 20 V higher than that of the specimen prepared in Na_3_PO_4_ alone.

The same test was conducted for the specimens prepared using Na_2_SiO_3_. In the case of Na_2_SiO_3_ alone, a current density of less than 40 A m^−2^ was observed at voltages of up to approximately 170 V (Fig. 11d). After reaching 170 V, the current increased rapidly. On the other hand, for the specimens prepared in Na_2_SiO_3_ with EG and GLY, a rapid current increase was observed only after reaching approximately 220 V. Thus, the withstand voltage clearly increased with the addition of a polyhydric alcohol (EG and GLY) into Na_2_SiO_3_-based electrolyte.

PEO films can be roughly divided into two parts: the inner layer and the outer layer. In general, the void sizes in the outer layer of PEO films are larger than those in the inner layer, indicating relatively greater compactness of the inner layer. Therefore, the present method was probably evaluating the compactness of the inner layer lying adjacent to the substrate. Weak spots in the PEO films likely allowed electrolyte solutions to migrate inward, resulting in substrate dissolution underneath the PEO films and subsequent rapid current increase during the voltage sweep. The tendency to increase withstand voltage by adding alcohol into basic electrolyte was confirmed even when the type of basic electrolyte was changed. In addition, highly corrosion-resistant films presented in Fig. 10 indicated high withstand voltage. Adding alcohol not only reduced the surface roughness of the PEO films due to the leveling action but was also effective in improving film properties (*e.g.*, withstand voltage, compactness, and corrosion resistance) by reducing structural defects (including pores, voids and cracks) in the PEO films. Although there is room for further investigation, the present approach is considered to constitute a valid method for the formation of compact PEO films with high corrosion resistance.

## 4 Conclusions

This research explored the effect of alcohol addition (*e.g.*, EtOH, EG, and GLY) to a Na_3_PO_4_-based electrolyte on the formation behavior, structure, and various properties of the PEO films formed on AZ31B magnesium alloy. Na_2_SiO_3_-based electrolyte was also used for comparison. The following conclusions were obtained as a result.

(1) The initial bend voltage and the steady-state oscillation voltage were both strongly dependent on electrolyte composition, both increasing with the addition of alcohol into the basic electrolyte, except for EtOH addition.

(2) For a given amount of electricity supplied, the thickness and corrosion resistance of PEO films were increased by the addition of EG and GLY. PEO films prepared in Na_3_PO_4_ containing EG or GLY were thicker than films prepared in Na_3_PO_4_ alone. In addition, surface roughness was effectively reduced by the addition of EG or GLY compared with that of films prepared in Na_3_PO_4_ alone.

(3) EG or GLY used as an additive could act not only as a leveler for the formation of compact film but also as an enhancer for film qualities (*e.g.*, corrosion resistance and withstand voltage), resulting in the formation of the PEO films with fewer structural defects.

## Conflicts of interest

There are no conflicts to declare.

## Supplementary Material

RA-010-D0RA01154A-s001

## References

[cit1] Song G. L., Atrens A. (1999). Corrosion mechanisms of magnesium alloys. Adv. Eng. Mater..

[cit2] Gray J. E., Luan B. (2002). Protective coatings on magnesium and its alloys—a critical review. J. Alloys Compd..

[cit3] Song G. L., Atrens A. (2003). Understanding magnesium corrosion—a framework for improved alloy performance. Adv. Eng. Mater..

[cit4] Shi Z., Song G., Atrens A. (2006). Corrosion resistance of anodised single-phase Mg alloys. Surf. Coat. Technol..

[cit5] Song Y. W., Shan D. Y., Han E. H. (2007). Corrosion behaviors of electroless plating Ni–P coatings deposited on magnesium alloys in artificial sweat solution. Electrochim. Acta.

[cit6] Atrens A., Song G., Cao F., Shi Z., Bowen P. K. (2013). Advances in Mg corrosion and research suggestions. J. Magnesium Alloys.

[cit7] Shi Z., Song G., Atrens A. (2006). The corrosion performance of anodised magnesium alloys. Corros. Sci..

[cit8] Vladimirov B. V., Krit B. L., Lyudin V. B., Morozova N. V., Rossiiskaya A. D., Suminov I. V., Epel’feld A. V. (2014). Microarc oxidation of magnesium alloys: a review. Surface Engineering and Applied Electrochemistry.

[cit9] Darband G. B., Aliofkhazraei M., Hamghalam P., Valizade N. (2017). Plasma electrolytic oxidation of magnesium and its alloys: mechanism, properties and applications. J. Magnesium Alloys.

[cit10] Bonilla F. A., Berkani A., Liu Y., Skeldon P., Thompson G. E., Habazaki H., Shimizu K., John C., Stevens K. (2002). Formation of anodic films on magnesium alloys in an alkaline phosphate electrolyte. J. Electrochem. Soc..

[cit11] Arrabal R., Matykina E., Viejo F., Skeldon P., Thompson G. E. (2008). Corrosion resistance of WE43 and AZ91D magnesium alloys with phosphate PEO coatings. Corros. Sci..

[cit12] Lv G.-H., Chen H., Wang X.-Q., Pang H., Zhang G.-L., Zou B., Lee H.-J., Yang S.-Z. (2010). Effect of additives on structure and corrosion resistance of plasma electrolytic oxidation coatings on AZ91D magnesium alloy in phosphate based electrolyte. Surf. Coat. Technol..

[cit13] Srinivasan P. B., Liang J., Balajeee R. G., Blawert C., Störmer M., Dietzel W. (2010). Effect of pulse frequency on the microstructure, phase composition and corrosion performance of a phosphate-based plasma electrolytic oxidation coated AM50 magnesium alloy. Appl. Surf. Sci..

[cit14] Birss V., Xia S., Yue R., Rateick Jr R. G. (2004). Characterization of oxide films formed on Mg-based WE43 alloy using AC/DC anodization in silicate solutions. J. Electrochem. Soc..

[cit15] Hsiao H.-Y., Tsung H.-C., Tsai W.-T. (2005). Anodization of AZ91D magnesium alloy in silicate-containing electrolytes. Surf. Coat. Technol..

[cit16] Duan H., Yan C., Wang F. (2007). Growth process of plasma electrolytic oxidation films formed on magnesium alloy AZ91D in silicate solution. Electrochim. Acta.

[cit17] Cai Q., Wang L., Wei B., Liu Q. (2006). Electrochemical performance of microarc oxidation films formed on AZ91D magnesium alloy in silicate and phosphate electrolytes. Surf. Coat. Technol..

[cit18] Ghasemi A., Raja V. S., Blawert C., Dietzel W., Kainer K. U. (2010). The role of anions in the formation and corrosion resistance of the plasma electrolytic oxidation coatings. Surf. Coat. Technol..

[cit19] Mori Y., Koshi A., Liao J., Asoh H., Ono S. (2014). Characteristics and corrosion resistance of plasma electrolytic oxidation coatings on AZ31B Mg alloy formed in phosphate–silicate mixture electrolytes. Corros. Sci..

[cit20] Guo H. F., An M. Z. (2005). Growth of ceramic coatings on AZ91D magnesium alloys by micro-arc oxidation in aluminate–fluoride solutions and evaluation of corrosion resistance. Appl. Surf. Sci..

[cit21] Duan H., Yan C., Wang F. (2007). Effect of electrolyte additives on performance of plasma electrolytic oxidation films formed on magnesium alloy AZ91D. Electrochim. Acta.

[cit22] Wang L., Chen L., Yan Z., Wang H., Peng J. (2009). Effect of potassium fluoride on structure and corrosion resistance of plasma electrolytic oxidation films formed on AZ31 magnesium alloy. J. Alloys Compd..

[cit23] Němcová A., Skeldon P., Thompson G. E., Pacal B. (2013). Effect of fluoride on plasma electrolytic oxidation of AZ61 magnesium alloy. Surf. Coat. Technol..

[cit24] Lim T. S., Ryu H. S., Hong S.-H. (2012). Electrochemical corrosion properties of CeO_2_-containing coatings on AZ31 magnesium alloys prepared by plasma electrolytic oxidation. Corros. Sci..

[cit25] Lu X., Mohedano M., Blawert C., Matykina E., Arrabal R., Kainer K. U., Zheludkevich M. L. (2016). Plasma electrolytic oxidation coatings with particle additions–a review. Surf. Coat. Technol..

[cit26] Zhao J., Xie X., Zhang C. (2017). Effect of the graphene oxide additive on the corrosion resistance of the plasma electrolytic oxidation coating of the AZ31 magnesium alloy. Corros. Sci..

[cit27] Asoh H., Matsuoka S., Sayama H., Ono S. (2010). Anodizing under sparking of AZ31B magnesium alloy in Na_3_PO_4_ electrolyte. J. Jpn. Inst. Light Met..

[cit28] Anawati, Asoh H., Ono S. (2015). Enhanced uniformity of apatite coating on a PEO film formed on AZ31 Mg alloy by an alkali pretreatment. Surf. Coat. Technol..

[cit29] Ono S., Moronuki S., Mori Y., Koshi A., Liao J., Asoh H. (2017). Effect of electrolyte concentration on the structure and corrosion resistance of anodic films formed on magnesium through plasma electrolytic oxidation. Electrochim. Acta.

[cit30] Lee K. M., Ko Y. G., Shin D. H. (2014). Microstructural characteristics of oxide layers formed on Mg–9 wt%Al–1 wt%Zn alloy via two-step plasma electrolytic oxidation. J. Alloys Compd..

[cit31] Einkhah F., Lee K. M., Sani M. A. F., Yoo B., Shin D. H. (2014). Structure and corrosion behavior of oxide layer with Zr compounds on AZ31 Mg alloy processed by two-step plasma electrolytic oxidation. Surf. Coat. Technol..

[cit32] Malayoglu U., Tekin K. C., Shrestha S. (2010). Influence of post-treatment on the corrosion resistance of PEO coated AM50B and AM60B Mg alloys. Surf. Coat. Technol..

[cit33] Narayanan T. S. N. S., Lee M. H. (2016). A simple strategy to modify the porous structure
of plasma electrolytic oxidation coatings on magnesium. RSC Adv..

[cit34] Phuong N. V., Fazal B. R., Moon S. (2017). Cerium- and phosphate-based sealing treatments of PEO coated AZ31 Mg alloy. Surf. Coat. Technol..

[cit35] Asoh H., Ono S. (2003). Anodizing of magnesium in amine-ethylene glycol electrolyte. Mater. Sci. Forum.

[cit36] Asoh H., Matsumoto M., Hashimoto H. (2019). Effects of ethanol on the efficiency of the formation of anodic alumina in sulfuric acid. Surf. Coat. Technol..

[cit37] Matsumoto M., Hashimoto H., Asoh H. (2020). Formation efficiency of anodic porous alumina in sulfuric acid containing alcohol: comparison of the effects of monohydric and polyhydric alcohols as additives. J. Electrochem. Soc..

[cit38] Akerlof G. (1932). Dielectric constants of some organic solvent-water mixtures at various temperatures. J. Am. Chem. Soc..

[cit39] Apelblat A. (2002). Dissociation constants and limiting conductances of organic acids in water. J. Mol. Liq..

[cit40] Boinet M., Verdier S., Maximovitch S., Dalard F. (2005). Plasma electrolytic oxidation of AM60 magnesium alloy: monitoring by acoustic emission technique. Electrochemical properties of coatings. Surf. Coat. Technol..

[cit41] Verdier S., Boinet M., Maximovitch S., Dalard F. (2005). Formation, structure and composition of anodic films on AM60 magnesium alloy obtained by DC plasma anodizing. Corros. Sci..

[cit42] Zhao L., Cui C., Wang Q., Bu S. (2010). Growth characteristics and corrosion resistance of micro-arc oxidation coating on pure magnesium for biomedical applications. Corros. Sci..

[cit43] Yerokhin A. L., Nie X., Leyland A., Matthews A., Dowey S. J. (1999). Plasma electrolysis for surface engineering. Surf. Coat. Technol..

[cit44] Wu D., Liu X., Lu K., Zhang Y., Wang H. (2009). Influence of C_3_H_8_O_3_ in the electrolyte on characteristics and corrosion resistance of the microarc oxidation coatings formed on AZ91D magnesium alloy surface. Appl. Surf. Sci..

[cit45] Qiu Z., Zhang Y., Li Y., Sun J., Wang R., Wu X. (2015). Glycerol as a leveler on ZK60 magnesium alloys during plasma electrolytic oxidation. RSC Adv..

[cit46] Hunter M. S., Fowle P. (1954). Factors affecting the formation of anodic oxide coatings. J. Electrochem. Soc..

