# Supplementary material for: Effect of alcohol addition on the structure and corrosion resistance of plasma electrolytic oxidation films formed on AZ31B magnesium alloy
Source: RSC Adv. 2020 Mar 2;10(15):9026–36. doi: 10.1039/d0ra01154a (PMC9050119; doi:10.1039/d0ra01154a)
Supplement: RA-010-D0RA01154A-s001 [file RA-010-D0RA01154A-s001.pdf]

Electronic Supplementary Material (ESI) for RSC Advances.
This journal is © The Royal Society of Chemistry 2020

Supporting Information

Effect of alcohol addition on the structure and corrosion resistance of plasma electrolytic oxidation films formed on AZ31B magnesium alloy

Hidetaka Asoh*, Kento Asakura, and Hideki Hashimoto

Department of Applied Chemistry, Kogakuin University,

2665-1 Nakano, Hachioji, Tokyo 192-0015, Japan

*Corresponding author. E-mail address: asoh@cc.kogakuin.ac.jp (H. Asoh).

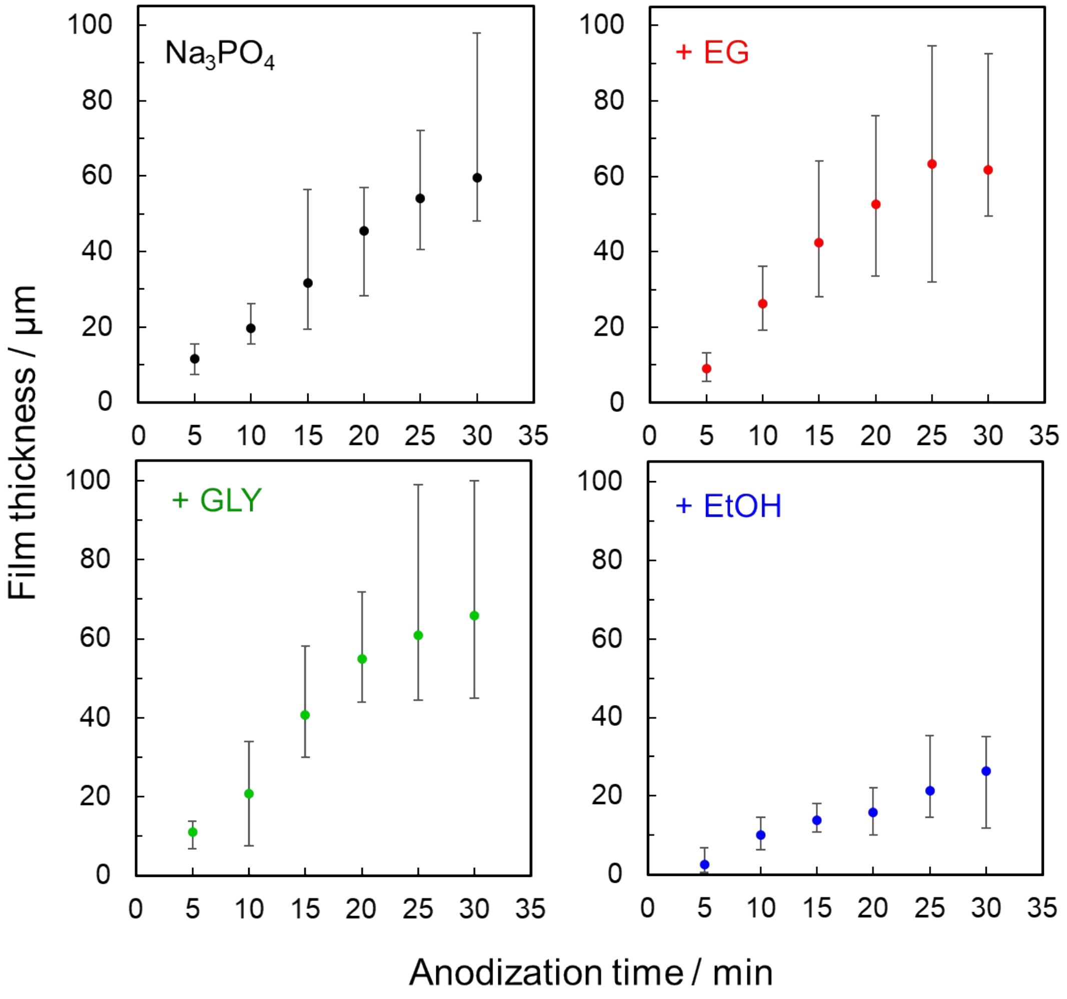

Figure S1 Thickness of the resulting PEO film as a function of anodization time. Anodization conditions were the same as those in Fig. 1(b). The thickness of the anodic films was measured using an eddy-current coating-thickness tester (Kett Electric Laboratory LH-373). The average value of 20 point measurements was used.

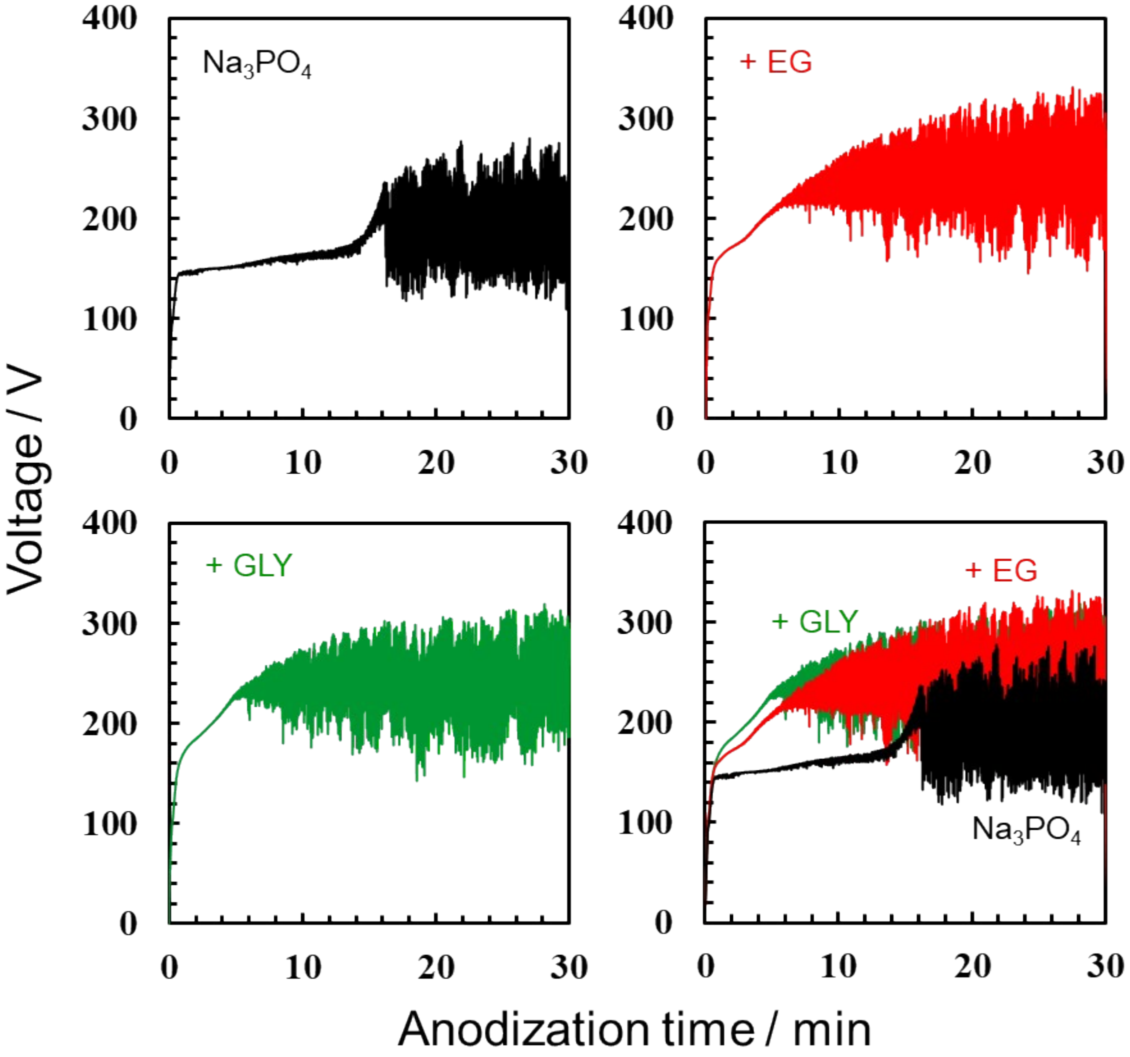

Figure S2 Voltage–time curves for the anodization of AZ31B magnesium alloy in 0.5 mol·dm^{-3} Na_3PO_4 with 5 vol.% various alcohols under a constant current density of 200 A·m^{-2} at 25 °C.

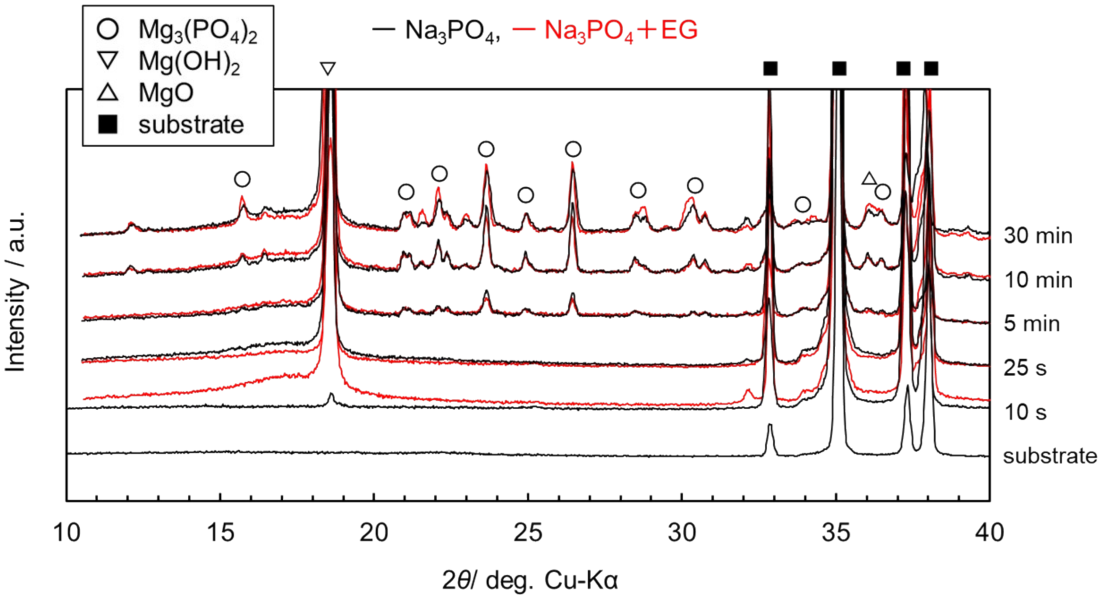

Figure S3 XRD patterns for the specimens after PEO treatment in 0.5 mol·dm^{-3} Na_3PO_4 with 5 vol.% EG addition under a constant current density of 200 A·m^{-2} at 25 °C for different anodization times.
